# Supplementary material for: Cisplatin is retained in the cochlea indefinitely following chemotherapy
Source: Nat Commun. 2017 Nov 21;8:1654. doi: 10.1038/s41467-017-01837-1 (PMC5698400; doi:10.1038/s41467-017-01837-1)
Supplement: Supplementary file 1 — Supplementary Information [file 41467_2017_1837_MOESM1_ESM.pdf]

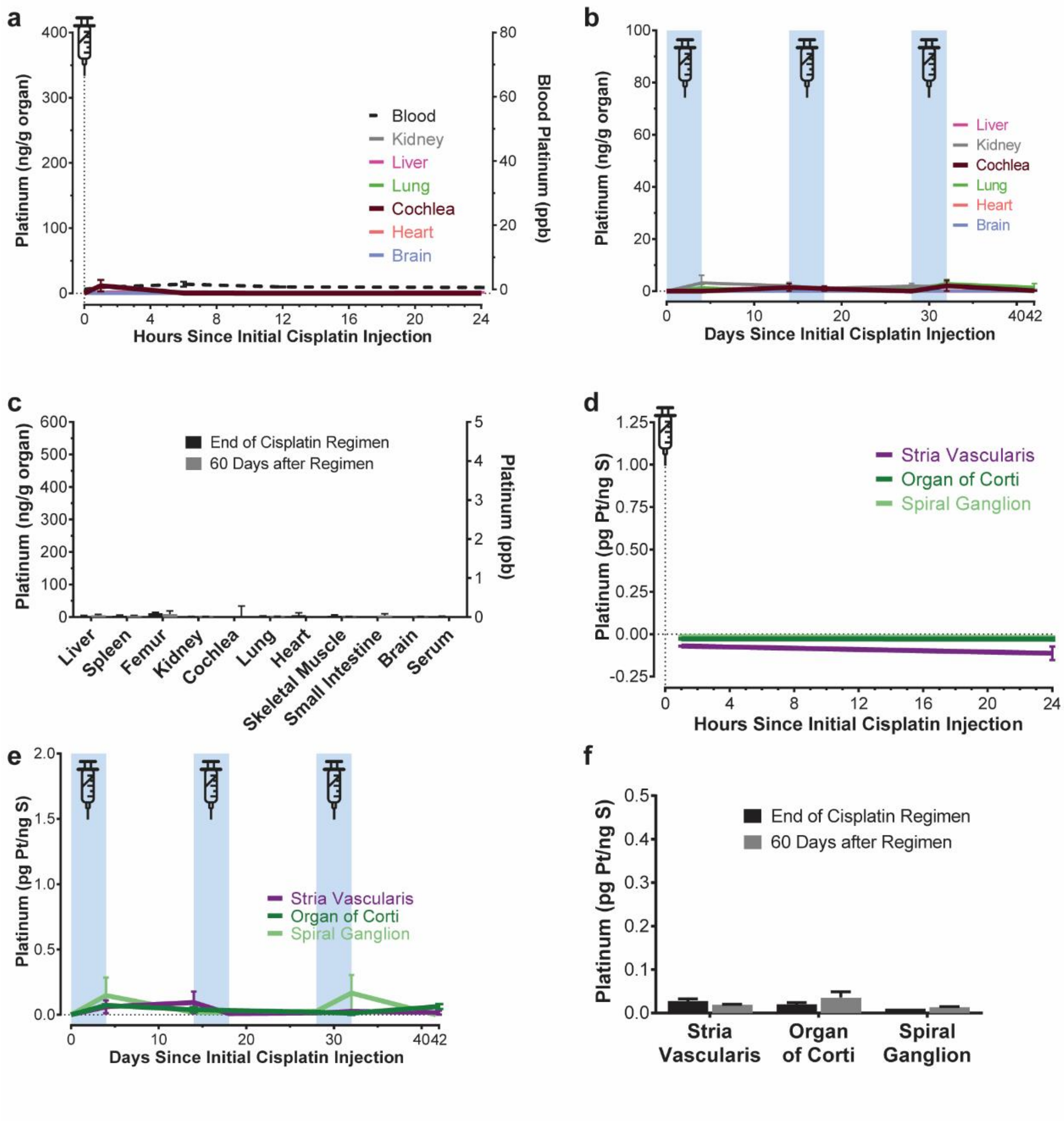

Supplementary Figure 1

Background measured concentrations of platinum are exceedingly low in all samples from saline-treated control mice. (a) Concentrations of platinum in isolated whole organs from the first 24 hours following a single i.p. injection of saline. Compare to Figure 2a. *n*=3 mice per time point. (b) Measured concentrations of platinum in whole mouse organs throughout the complete saline control regimen. Compare to Figure 2b. *n*=3 mice per time point. (c) Concentrations of platinum in whole mouse organs at the end of the saline regimen and following a subsequent 60-day recovery. Compare to Figure 2c. *n*=3-4 mice in each group. (d) Platinum measured in microdissected regions of the mouse cochlea during the first 24 hours following a single i.p. injection of saline. Compare to Figure 3b. *n*=3 mice at each time point. (e) Concentrations of platinum in microdissected regions of the mouse cochlea throughout the complete saline control regimen. Compare to Figure 3c. *n*=3 mice at each time point. (f) Concentrations of platinum in cochlear regions at the end of the saline regimen and following a subsequent 60-day recovery. Compare to Figure 3d. *n*=4-7 mice.

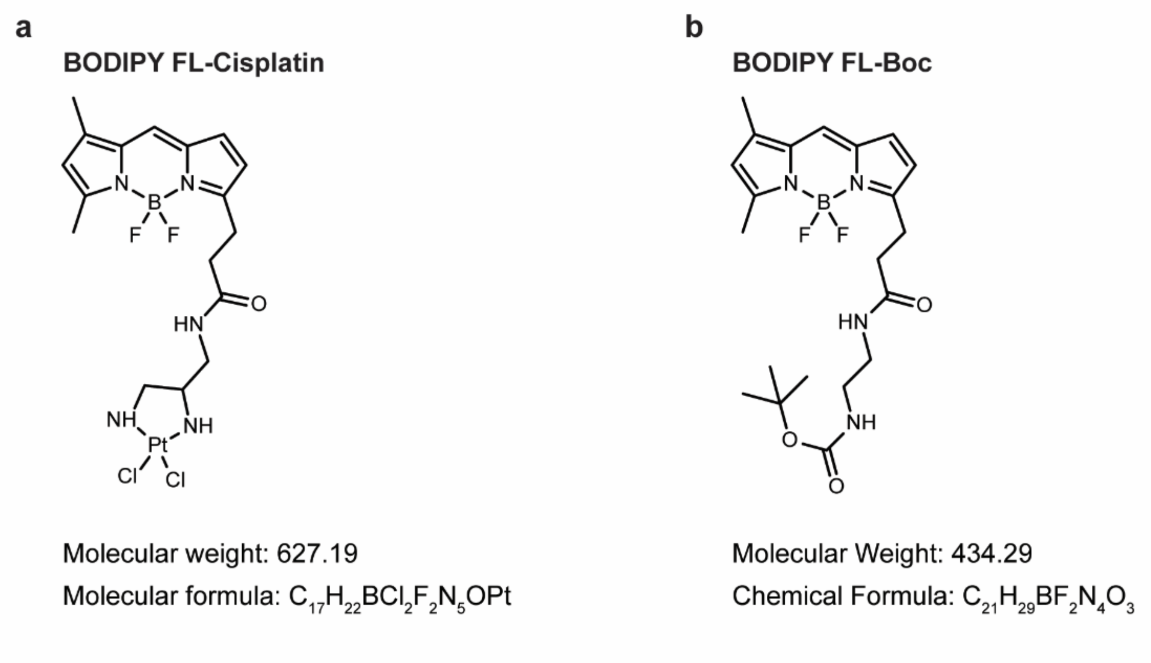

Supplementary Figure 2

BODIPY FL-Cisplatin is a small fluorophore conjugate which can be used for visualization of cisplatin trafficking. (a) The BODIPY FL-Cisplatin molecule is a validated conjugate recently shown to retain DNA damaging and cytotoxicity capacity [19,20]. (b) The free-dye control (no cisplatin) compound BODIPY FL-Boc was used to control for any observed BODIPY FL-Cisplatin pharmacokinetics that may have been a result of the properties of the fluorophore and not of the cisplatin moiety.

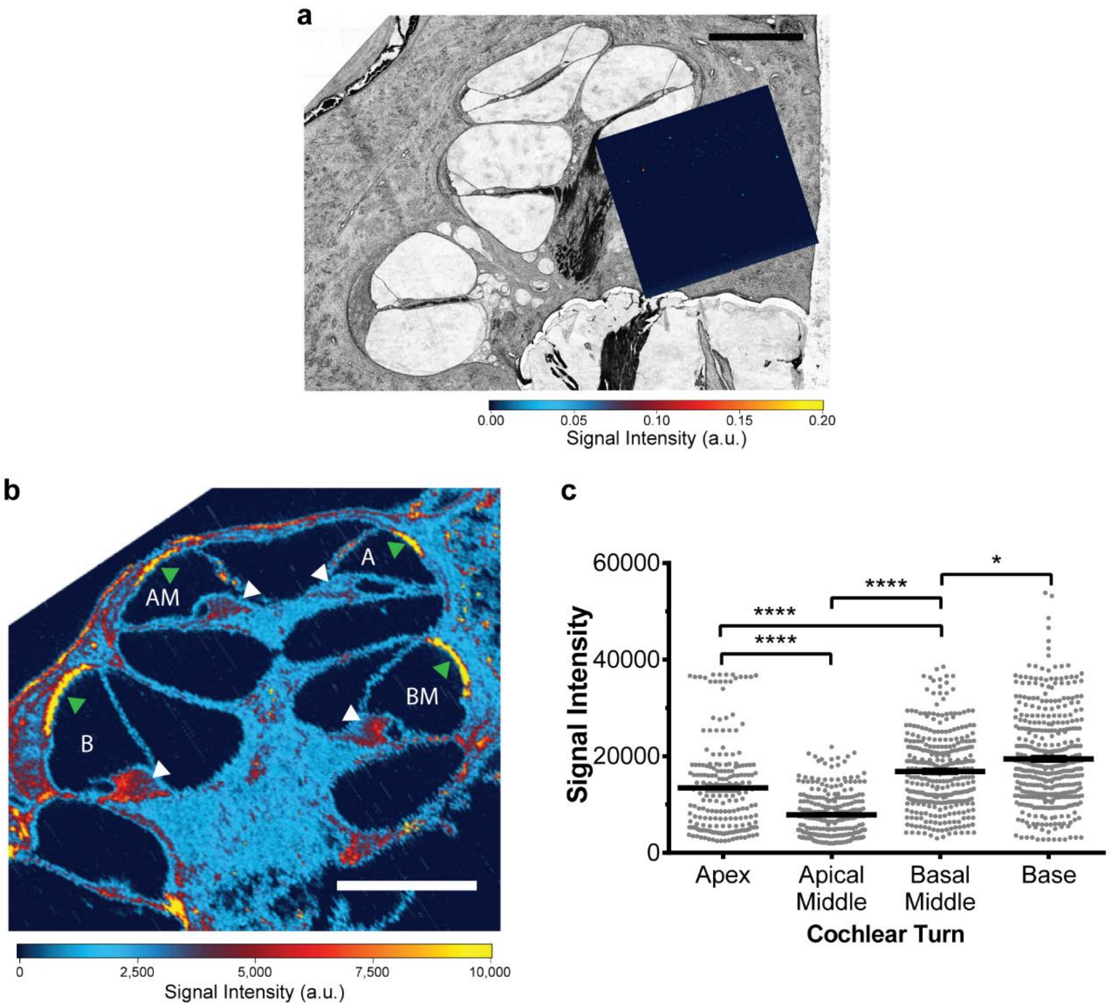

Supplementary Figure 3

Laser ablation ICP-MS images of platinum distribution in human and murine cochlear sections. (a) Representative laser ablation ICP-MS overlay of platinum distribution throughout a cochlear section from a cisplatin-naïve control patient. The LA ICP-MS is overlaid upon pre-ablation brightfield image of the cochlear section. Scale bar = 1mm. a.u. = arbitrary units. (b) Laser ablation ICP-MS image of platinum distribution throughout the cochlea of a cisplatin-treated mouse. Platinum signal intensity in the spiral limbus (white arrowheads) appears to decrease from cochlear base to apex. Green arrowheads mark stria vascularis in each cochlear turn. Cochlear turns are marked: A = apex, AM = apical middle, BM = basal middle, and B = base. Scale bar = 500 µm. (c) Platinum signal intensity values for each pixel along a linear region of interest inside the stria vascularis at different cochlear turns. Average platinum intensity values significantly increase from apex to base. Values were obtained from the single ablation shown in panel b. The Kruskal-Wallis test followed by Dunn's multiple comparisons test was used to determine significance. Data are expressed as mean ± s.e.m. * $P<0.05$, **** $P<0.0001$.
